# Refractory plasmonics enabling 20% efficient lead-free perovskite solar cells

**DOI:** 10.1038/s41598-020-63745-7

**Published:** 2020-04-21

**Authors:** Ahmed A. Mohsen, Mohamed Zahran, S. E. D. Habib, Nageh K. Allam

**Affiliations:** 1 Energy Materials Laboratory (EML), School of Sciences and Engineering, The American University in Cairo, New Cairo, 11835 Egypt; 2 Nanotechnology Laboratory, Electronics Research Institute, Cairo, Egypt; 3 Electronics and Communications, Faculty of Engineering, Cairo University, Giza, Egypt

**Keywords:** Devices for energy harvesting, Solar cells

## Abstract

Core-shell refractory plasmonic nanoparticles are used as excellent nanoantennas to improve the efficiency of lead-free perovskite solar cells (PSCs). SiO_2_ is used as the shell coating due to its high refractive index and low extinction coefficient, enabling the control over the sunlight directivity. An optoelectronic model is developed using 3D finite element method (FEM) as implemented in COMSOL Multiphysics to calculate the optical and electrical parameters of plain and ZrN/SiO_2_-modified PSCs. For a fair comparison, ZrN-decorated PSCs are also simulated. While the decoration with ZrN nanoparticles boosts the power conversion efficiency (PCE) of the PSC from 12.9% to 17%, the use of ZrN/SiO_2_ core/shell nanoparticles shows an unprecedented enhancement in the PCE to reach 20%. The enhancement in the PCE is discussed in details.

## Introduction

The promising efficiency and affordable cost are the driving forces pushing the research on lead (Pb)-based perovskite solar cells (PSCs) over the past decade^1–5^. The latest power conversion efficiency (PCE) certified by the National Renewable Energy Laboratory (NREL) for PSCs is 24.2% as reported by KRICT/MIT^6^, competing that of Si-based photovoltaic and surpassing CdTe solar cells^7,8^. However, the toxicity concerns of Pb were proved to be a serious flaw and can hurdle the path of PSCs towards commercialization^9–12^. Partial replacement of Pb with lower toxicity cations has been reported and proposed as Ge^13^, Sb^14^, and Sn^15,16^. Among those candidates, Sn has shown a close similarity to Pb^17^ with a remarkable enhancement in PCE from 6%^16^ to 10%^18^. However, this PCE is much lower than that reported for Pb-based PSCs, leaving a room for improvement. Generally, the lack of light absorption is the major concern hindering the achievement of high PCE for thin film solar cells (TFSCs) due to the small thickness of the active layer used. In this regard, the use of plasmonic light trapping nanoparticles has emerged as a means to enhance the PCE of TFSCs^19–22^. Plasmonic nanoparticles act as nanoantennas that couple near field electromagnetic waves (sunlight) to sub-wavelength area, enhancing the nanoscale light-material interactions^23–25^. The geometry, dielectric permittivity, surface roughness, and surrounding medium are the factors determining the plasmonic near-field intensity. To this end, noble metal nanoparticles (Au, Ag, etc.) have been reported to enhance the efficiency of TFSCs^26,27^ due to their ability to couple electromagnetic waves at air/metal interactions using surface plasmon guided modes and control the direction of scattered light^28,29^. However, noble metals are expensive, exhibit narrow-band scattering and absorption spectra, thermally unstable, and CMOS incompatible. As alternatives, refractory plasmonic metal nitrides such as TiN and ZrN have been investigated with promising results being reported that were ascribed to their highly tunable optoelectronic characteristics^30–34^ Of special interest, plasmonic/dielectric core/shell nanostructures have attracted recent attention mainly due to the fact that dielectrics are transparent to sunlight and enjoy high refractive index that enables the control over light coupling^35^ and the possibility of tuning the surface plasmon resonance^36^. The use of core-shell structure provides both electrical and chemical protection isolation to the plasmonic core as it prevents the metal diffusion contaminant into the solar cell structure.

Herein, we show the opportunity to boost the efficiency of Pb-free perovskite solar cells (PSCs) to reach up to 20% upon the implantation of ZrN/SiO_2_ core/shell nanoparticles in the active layer. For comparison, ZrN and TiN-decorated PSCs have also been investigated and discussed.

## Optoelectronic Modeling Details

**Figure 1:**
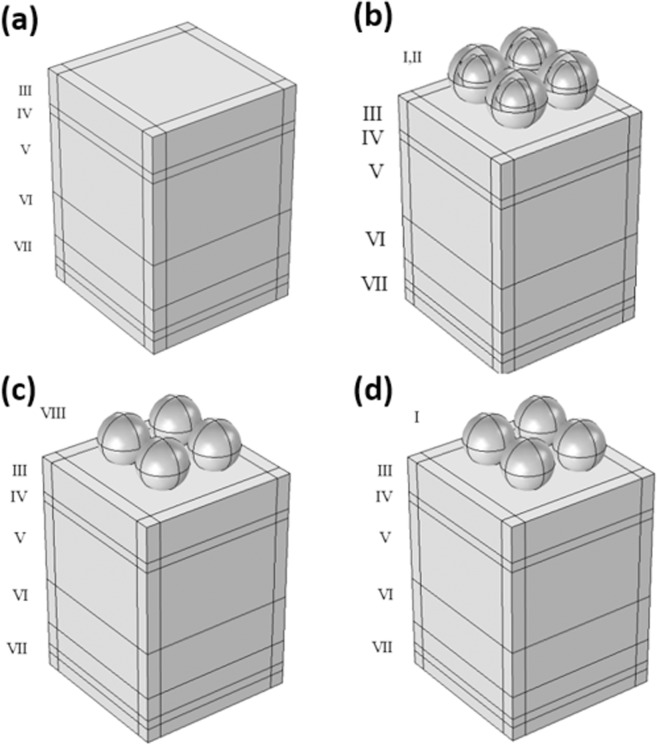
(**a**) Planar perovskite solar cell structure consisting of (VII) gold (Au) as a back contact with a thickness of 100 nm, (VI) HTM (Spiro–OMeTAD) with a thickness of 200 nm, (V) MASnI_3_ active material with a thickness 350 nm, (IV) ETM (TiO_2_) with thickness of 40 nm, and (III) ITO with thickness of 150 nm. (**b**) planar perovskite with the core-shell nanoparticles consisting of (I) ZrN nanoparticles as a plasmonic core and shielded by (II) SiO_2_ nanoshells with thickness of 40 nm. (**c**) planar perovskite solar cell implanted above ITO are the (VIII) TiN nanoparticles. (**d**) planar perovskite solar cell implanted above ITO are the (I) ZrN nanoparticles, the proposed solar cell structure is of width and depth of 600 nm.

Four 3D electromagnetic wave (EMW) models have been constructed based on finite element method (FEM). The first model is used to calculate the interaction of sunlight electromagnetic radiation (AM 1.5 G) with solar cell active layers based on Maxwell’s equations of light propagation:

1 $$\frac{\partial H}{\partial t}=\frac{-1}{\mu }\nabla \times E$$

2 $$\varepsilon \frac{\partial E}{\partial t}=\nabla \times H-\sigma E$$

where *H* is the magnetic field, *E* is the electric field, *μ* is the permeability, ℇ is the electric permittivity, and σ is the electric conductivity. This model is used to develop the full field of the proposed design in Fig. 1a of planar perovskite solar cell in order to obtain the generation rate and short circuit current (*Jsc*). The optical carrier generation rate (*G*_*opt*_) per wavelength over the active layer volume is calculated using Eq. 3 ^37^.

3 $${G}_{opt}(\lambda )=\frac{\varepsilon {\prime\prime} {|E|}^{2}}{2\hslash }$$

where the optical carrier generation depends on the complex permittivity imaginary part (*ε*″) and the electric field intensity. Upon integrating the optical carrier generation over the simulated wavelength (*λ*), the total generation rate (TGR) can be estimated using Eq. 4.

4 $$TGR={\int }_{{\lambda }_{1}}^{{\lambda }_{2}}{G}_{opt}(\lambda )d\lambda $$

where *λ*_1_ and *λ*_2_ are the minimum and maximum of simulated wavelengths that have been swept over the optical model, respectively. The second and third models are based on coupling the optoelectrical model with the first model as we used the full field of the planar model as an input to simulate the nanostructured refractory plasmonics model. TiN and ZrN nanoparticles were used as nanoscatterers with different radii (50 nm, 75 nm, 100 nm) on the top of the Sn-based perovskite solar cell and compare the emerged plasmon enhancements with respect to the planar model. The proposed design of TiN and ZrN nanoparticles are shown in Fig. 1b,c. The normalized light absorption profile was calculated using Eq. 5 ^38^.

5 $$Normalized\,Absorption=\frac{Absorption\,of\,nanostructured\,film}{Absorption\,of\,planar\,film}$$

The fourth model proposes a novel design of ZrN nanoparticles with different radii (50 nm, 75 nm) as a plasmonic core and SiO_2_ as a dielectric shell of thickness 40 nm. The proposed design is shown in Fig. 1d.

The external quantum efficiency (*EQE*) was calculated for the planar, TiN, and ZrN nanostructured solar cells using Eq. 6 ^38^ assuming single-path absorption and unit internal quantum efficiency (*IQE*).

6 $$\text{EQE}=\text{IQE}\,\ast \,\text{Absorption}$$

The optical refractive indices and extinction coefficients of Au, MASnI_3_, TiO_2_, Spiro-OMeTAD, ITO, TiN, and ZrN were taken from previously published data^39–45^. The electrical model was based on the use of the *G*_*opt*_ as an input parameter. The current-voltage characteristics were obtained by solving the drift-diffusion and 3D Poisson’s equations. Series and Shunt resistances were used as fitting parameters from previously measured data^46^. The *J*_*sc*_ was calculated using Eq. 7 ^47^.

7 $${J}_{sc}=q{\int }_{{\lambda }_{1}}^{{\lambda }_{2}}\frac{AM\,1.5G(\lambda )\times (1-\exp (-\alpha d))}{\frac{hc}{\lambda }}d\lambda $$

where *q* is the elementary charge, *d* is the thickness of the active layer, and *α* is the absorption coefficient calculated using Eq. 8.

8 $$\alpha (\lambda )=\frac{4\pi k(\lambda )}{\lambda }$$

where *k(λ)* is the extinction coefficient for each simulated wavelength. As *J*_*sc*_ is directly proportional to the total carrier generation rate and have the same dimensions of the planar model, Eq. 9 can be used to calculate any enhancement in *J*_*sc*_^48^.

9 $${J}_{sc}\text{enhancement}=\frac{Total\,Generation\,Rate\,using\,nanostructures}{Total\,Generation\,Rate\,of\,planar\,cell}$$

The COMSOL Multiphysics software package was used with AM 1.5 G as an input power port and a wavelength in the range 100–1300 nm was swept over with 10 nm step size in order to cover the UV, visible, and IR regions to obtain the greatest possible plasmonic enhancement over the whole possible simulated bandwidth because TiN and ZrN nanoparticles have an increasing extinction coefficient in the IR bandwidth^44,45^. The optoelectrical model was applied on a cell surrounded by perfectly matched layers (PMLs) in all directions and periodic boundary conditions (PBCs) in x-y direction. However, there were some hurdles in the optical model. First, the largest value allowed for mesh size must not exceed λ/10 of the smallest simulated wavelength. Therefore, a tetrahedron shape was selected for meshing the solar cell active layers, where the smaller the mesh size, the more accurate the computational data compared to the practical counterpart. However, this comes on the expense of the computational time. Second, in order to decrease the computational time, a mapped meshing with normal or coarse meshing was applied to the PMLs surrounding the solar cell in order to allow the use of smaller mesh size for the solar cell and the active material. Third, the *G*_*opt*_ was integrated to the full simulated bandwidth and used as an input data profile to construct the electrical model. In the electrical model, any enhancements in light absorption was assumed to be directly proportional to the enhancements in the photocurrent as shown by Eq. 9. The electrical parameters of TiO_2_, MASnI_3_, and Spiro-OMeTAD were extracted from literature^49,50^.

## Results and discussion

### Nanoparticles-decorated PSCs

**Figure 2:**
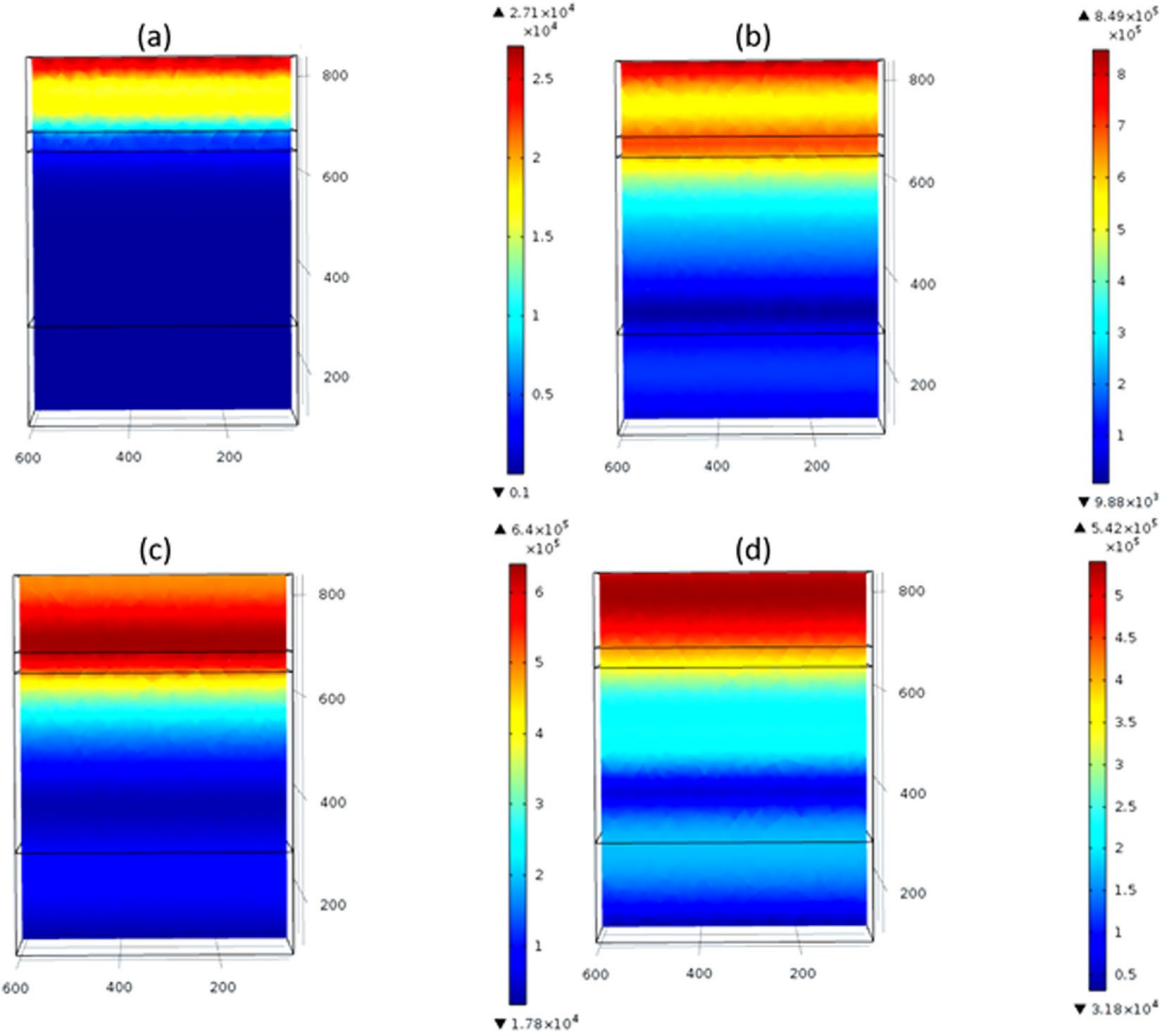
Electric field profiles at different wavelengths: (**a**) 300 nm, (**b**) 800 nm, (**c**) 1000 nm, and (d) 1200 nm for planar PSCs.

To construct the optoelectrical model of the TiN or ZrN-decorated PSCs, the optoelectrical model for plain (planar) solar cell is developed first to calculate the *G*_*opt*_ and *J*_*sc*_ for planar PSC for better comparison. The proposed design is shown in Fig. 1a, where the thicknesses of Au, Spiro-OMeTAD, MASnI_3_, TiO_2_, and ITO layers are 100 nm, 200 nm, 40 nm, 350 nm, and 150 nm, respectively with 600 nm width and 600 nm depth. The electric field profiles are investigated at selected wavelengths that represent the UV, visible, and IR bandwidths (i.e. full bandwidth scan). The electric field profiles of the planar perovskite solar cell are shown in Fig. 2.

#### TiN-decorated PSCs

**Figure 3:**
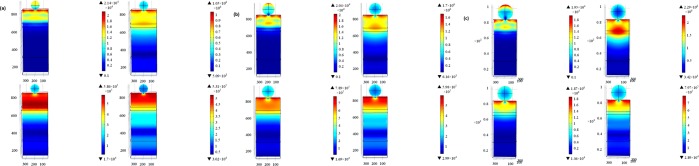
Electric field profiles at different wavelengths: (**a**) 300 nm, (**b**) 800 nm, (**c**) 1000 nm, and (id) 1200 nm for (**a**) 50 nm, (**b**) 75 nm, and (**c**) 100 nm TiN nanoparticles-decorated PSCs, respectively.

**Figure 4:**
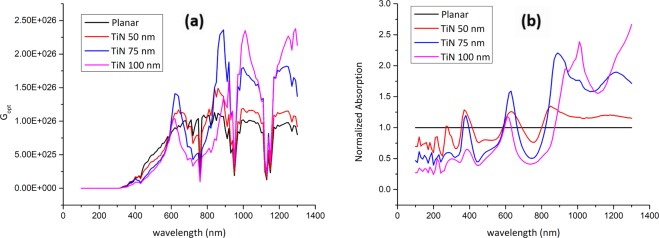
(**a**) Optical carrier generation rate (*G*_*opt*_) and (**b**) normalized light absorption profiles for planar and TiN-decorated PSCs.

TiN nanoparticles were implanted on-top of the PSC active layer as discussed in model 2 and shown in Fig. 1b. Figure 3 shows the electric field profiles upon implanting TiN nanoparticles and their electromagnetic radiation resonance coupling enhancement to the solar cell active layers, which glows the most in the IR range compared to the planar PSCs counterparts. For each simulated TiN nanoparticles’ size, the corresponding *G*_*opt*_ is calculated using Eq. 3 and plotted in Fig. 4a. TiN acts as an absorber in the bandwidth range 300 nm − 580 nm with the absorption coefficient being significantly decreasing at wavelengths greater than 780 nm. Note that the TiN nanoparticles that are 100 nm in radius have their plasmonic resonance peak at a wavelength of 880 nm, indicating the high scattering capability of TiN in the IR range^45^. While the 75 nm radius TiN nanoparticles showed two plasmonic resonance peaks at 1000 nm and 1280 nm, the 50 nm radius counterparts showed a resonance peak at 840 nm. The TGR is calculated using Eq. 4 and listed in Table 1, indicating a maximum enhancement of 36.7% for the PSC decorated with 75 nm TiN particles compared to the planar PSC counterpart.

**Table 1:** Optical and electrical parameters for planar and TiN-implanted PSCs.

Structure	*TGR* (m^−3^ s^−1^)	*TGR* Enhancement	*J*_*sc*_ (mA/cm^2^)	*V*_*oc*_ (mV)	*FF* (%)	*PCE* (%)
Planar	7.1840 × 10^27^	----	27	680.47	70.21	12.9
TiN (50 nm)	7.9038 × 10^27^	10.02%	29.71	683.50	70.64	14.3
TiN (75 nm)	9.225 × 10^27^	28.4%	34.7	687.57	71.23	17
TiN (100 nm)	9.8205 × 10^27^	36.7%	36.91	689.2	71.48	18.2

Figure 4b shows the normalized absorption calculated using Eq. 5 for the simulated PSCs. TiN nanoparticles function as nano-antennas with their surface plasmon resonance (SPR) modes coupled to the PSC active layers due to the increase of the near field sunlight in the IR region (> 800 nm). This absorption enhancement, especially in the IR bandwidth, due to the plasmonic nanoparticles effect to act as wave guide to direct sunlight by means of localized SPR, forming surface plasmon polaritons (SPP) at the air/TiN nanoparticle interface that couple sunlight into sub-wavelengths active area.

**Figure 5:**
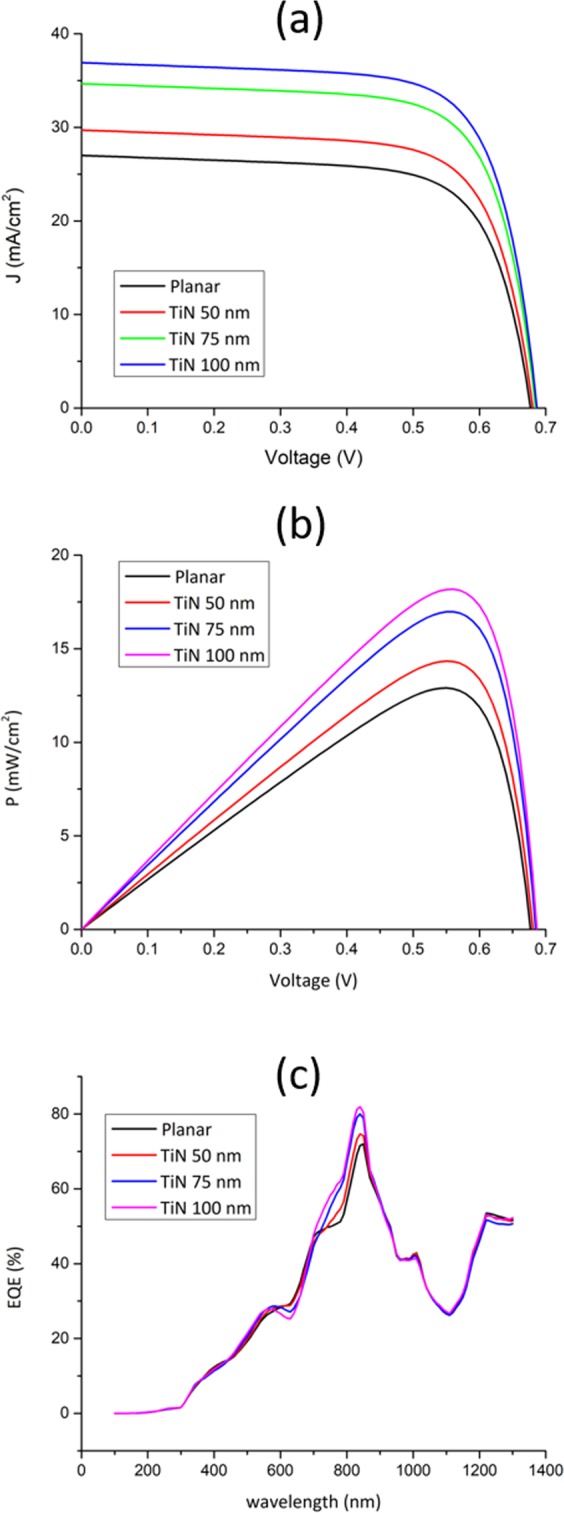
(a) *J-V*, (b) *P-V*, and *EQE* characteristics of the proposed PSCs.

The corresponding *J-V* and *P-V* characteristics of the simulated PSCs are shown in Fig. 5 and listed in Table 1. Note that the series and shunt resistances are tuned based on the reports in literature taking into account the recombination mechanisms in PSCs^46^. The optimized series resistance is found to be 50 Ω and the shunt resistance is 400 Ω. The *J*_*sc*_ shows a pronounced enhancement upon TiN-decoration compared to that of the planar PSC. The maximum enhancement in PEC is observed for the PSC decorated with the 100 nm TiN nanoparticles as it is increased from 12.9% for the planar PSC to 18.2%, achieving ca. 41% enhancement in the overall solar cell efficiency. Figure 5c shows the EQE enhancement for each of the TiN-decorated PSCs. Note that the 100 nm TiN has an EQE of 85% due its high electric field directivity as shown in Fig. 2, where the localized surface plasmonic resonance of TiN nanoparticles is shown at 840 nm, in agreement with Shalaev *et al*.^33^.

#### ZrN-decorated PSCs

**Figure 6:**
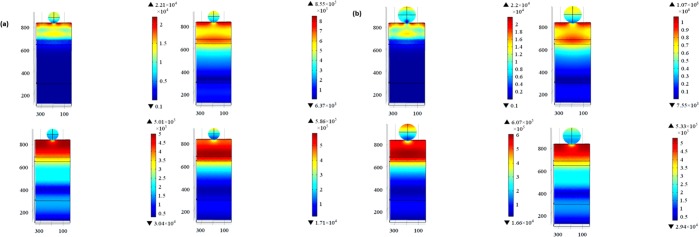
Electric field profiles of the ZrN-decorated PSCs: ZrN radii (**a**) 50 nm (**b**) 75 nm, and (**c**) 100 nm at wavelengths of 300 nm, 800 nm, 1000 nm, and 1200 nm, respectively.

**Figure 7:**
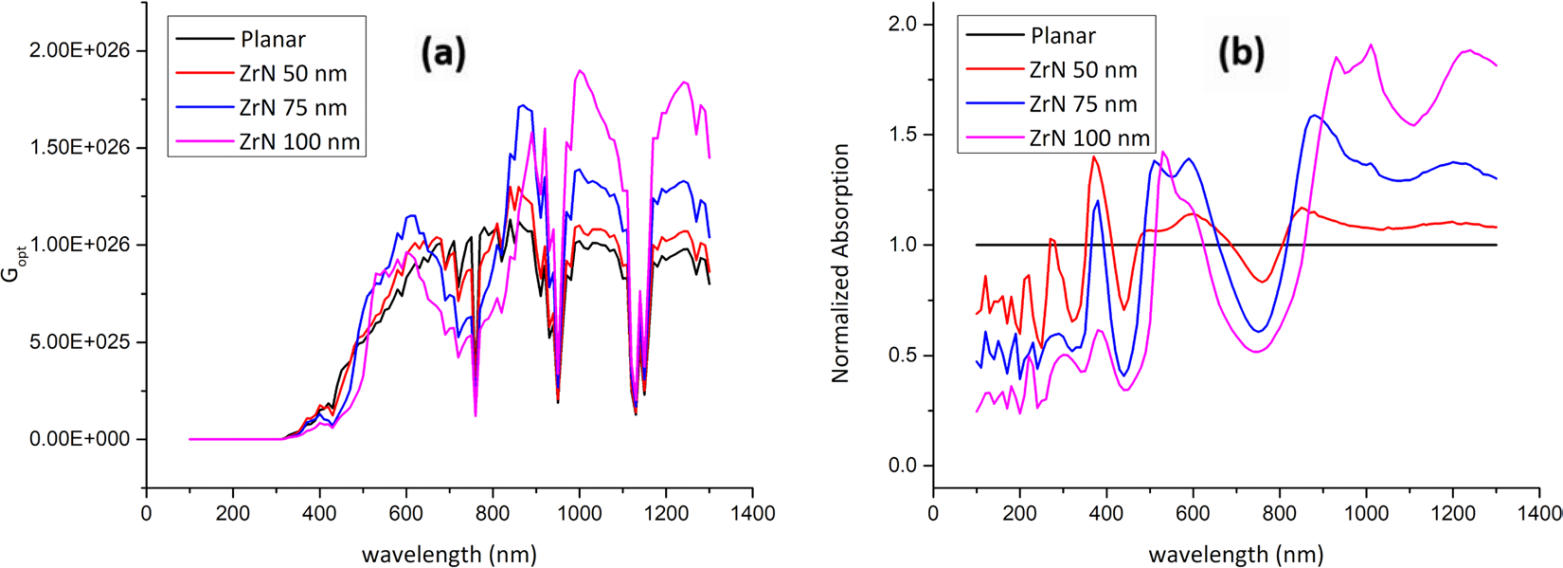
(**a**) Optical carrier generation rate (*G*_*opt*_) and (**b**) normalized light absorption profiles for planar and ZrN-decorated PSCs.

**Figure 8:**
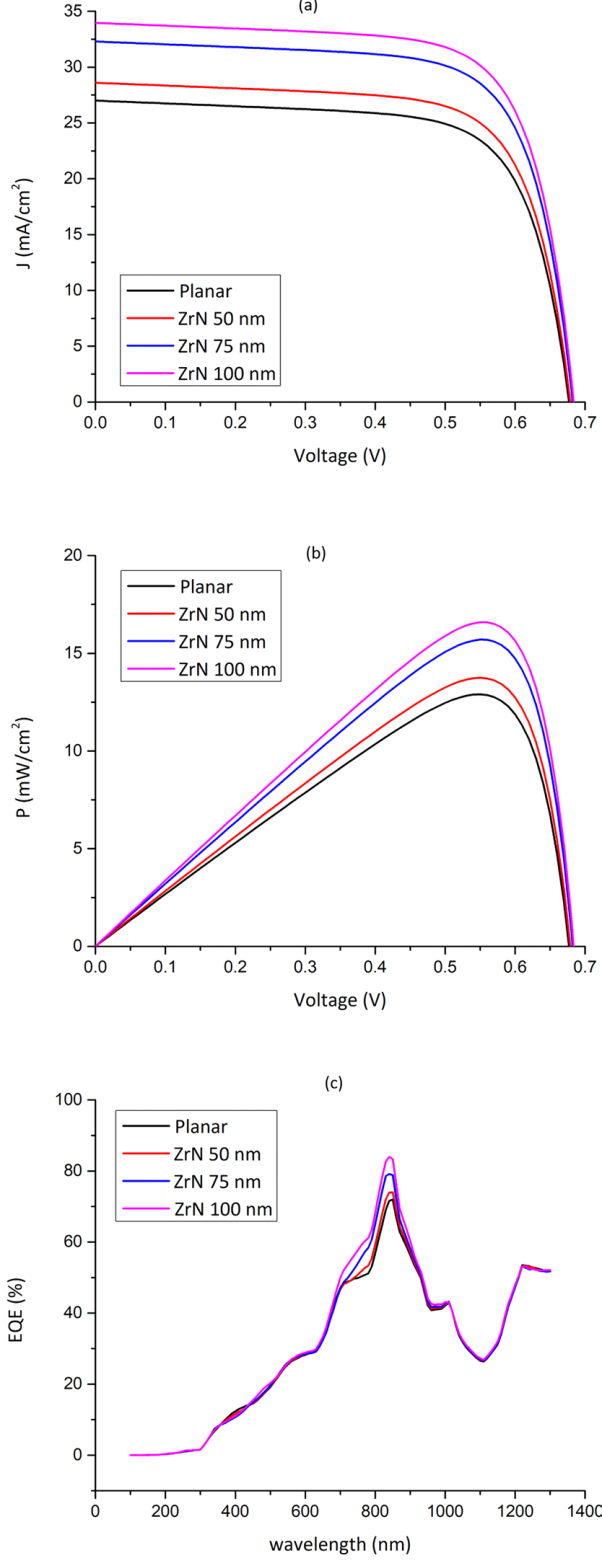
(**a**) *J-V*, (**b**) *P-V*, and *EQE* characteristics of the proposed PSCs.

ZrN is another very promising refractory plasmonic material. The electric field profile for ZrN nanoparticles implanted onto the active layer of PSCs is shown in Fig. 6. Also, the G_opt_ and the normalized optical absorption (NOA) are shown in Fig. 7. Note that the PSC decorated with 100 nm ZrN particles showed significant plasmonic resonance enhancement at the wavelengths of 1000 nm and 1250 nm. Upon integrating the G_opt_ over the swept simulated wavelength using Eq. 4, it was possible to calculate the total generation rate enhancement as listed in Table 2. Note that the 100 nm ZrN particles showed 25.8% enhancement in the TGR compared to planar PSC with an increase in the PCE from 12.9% (planar) to 16.6%. Moreover, a maximum EQE of 84.63% at 840 nm, compared to 72% for the planar PSC at the same wavelength, is observed, Fig. 8.

**Table 2:** The optical and electrical parameters of the ZrN-decorated PSCs.

Structure	TGR (m^−3^ s^−1^)	TGR Enhancement	J_sc_ (mA/cm^2^)	V_oc_ (mV)	FF (%)	PCE (%)
Planar	7.1840 × 10^27^	----	27	680.5	70.21	12.9
ZrN (50 nm)	7.6106 × 10^27^	5.9%	28.8	681.9	70.48	13.7
ZrN (75 nm)	8.5930 × 10^27^	19.6%	32.6	685.1	70.96	15.7
ZrN (100 nm)	9.0375 × 10^27^	25.8%	34.2	686.4	71.14	16.6

### Novel ZrN/SiO_2_ core/shell-decorated PSCs

**Figure 9:**
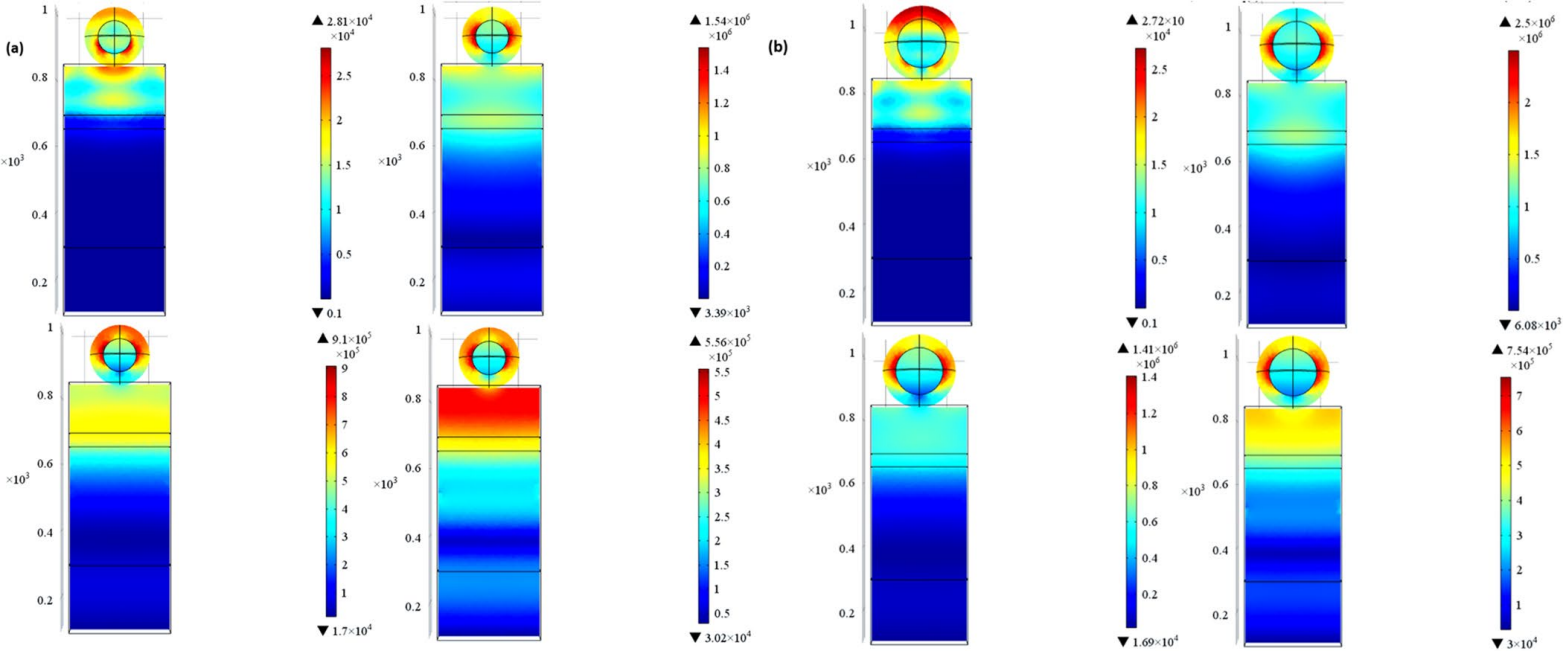
Electric field profiles of ZrN/SiO_2_ core-shell-decorated PSCs: (**a**) 90 nm (50 nm core surrounded by 40 nm shell) and (**b**) 115 nm (75 nm core surrounded by 40 nm shell) at wavelengths of 300 nm, 800 nm, 1000 nm and 1200 nm, respectively.

**Figure 10:**
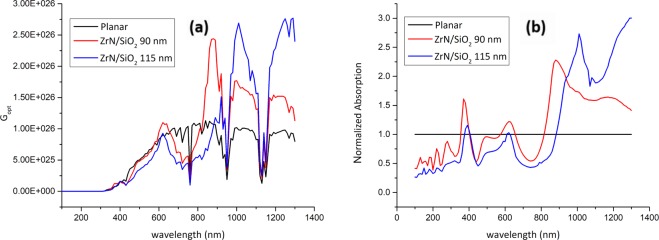
(**a**) Optical carrier generation rate (*G*_*opt*_) and (**b**) normalized light absorption profiles for planar and ZrN/SiO_2_-decorated PSCs.

**Figure 11:**
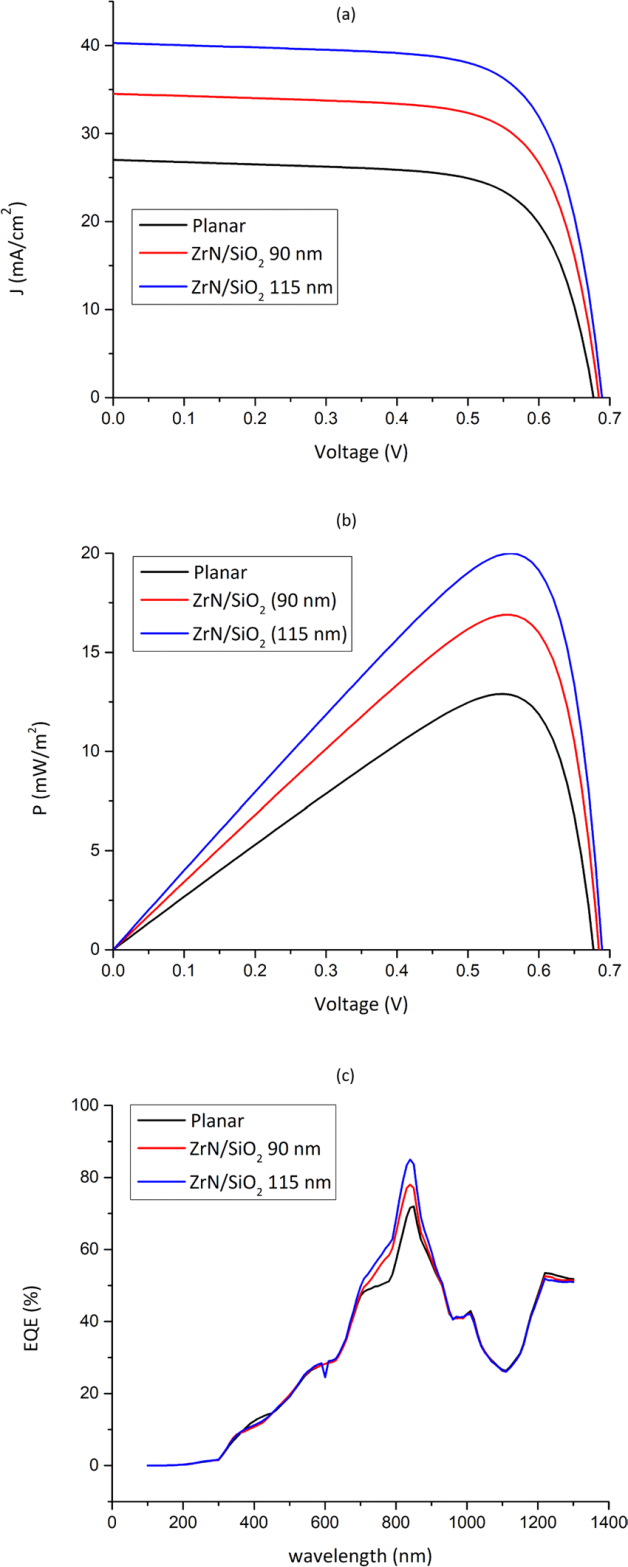
(**a**) *J-V*, (**b**) *P-V*, and (**c**) *EQE* characteristics of proposed ZrN/SiO_2_-decorated PSCs.

ZrN/SiO_2_ core/shell structure has been tested as a novel structure to enhance the efficiency of PSC using two different radii of ZrN of 50 nm and 75 nm core surrounded by 40 nm of silica (SiO_2_) shell. The core/shell structure is used as a means to tune the LSPR of ZrN, which depends on the surrounding dielectric medium. Figure 9 shows the electric field profiles, indicating a great enhancement in the sunlight coupled to the solar cell active layer. Figure 10 shows a tremendous enhancement in the *G*_*opt*_ and optical absorption in the active layers of the PSCs upon the use of ZrN/SiO_2_. Moreover, a 13.3 mA/cm^2^ increase in *J*_*sc*_, 55% enhancement in the *TGR*, and an increase in the *PCE* from 12.9% to 20% are observed as listed in Table 3 and Fig. 11a,b. The EQE is 87% at 840 nm as recorded for the PSC decorated with 115 nm ZrN/SiO_2_, see Fig. 11c.

**Table 3:** The optical and electrical parameters of the ZrN/SiO_2_-decorated PSCs.

Structure	TGR (m^−3^ s^−1^)	TGR Enhancement	J_sc_ (mA/cm^2^)	V_oc_ (mV)	FF (%)	PCE (%)
Planar	7.1840 ×10^27^	----	27	680.5	70.2	12.9
ZrN/SiO_2_ (90 nm)	9.1853 ×10^27^	31%	34.5	687.4	71.2	16.9
ZrN/SiO_2_ (115 nm)	1.0721 ×10^28^	55%	40.3	691.4	71.8	20

Table 4 summarizes the calculated optical and electrical parameters of the PSCs-decorated with different refractory plasmonic nanoarchitectures. Note that the generation rate of ZrN/SiO_2_ (75 nm core and 40 nm shell) increases by 19.8% and 15.7% compared to ZrN alone with 75 nm and 100 nm in radius, respectively. This can be ascribed to the enhancement in the plasmonic surface plasmon directivity by the dielectric shell.

**Table 4:** Comparison of optical and electrical parameters upon the use of different nanostructures.

Parameters	TGR (m^−3^ s^−1^)	J_sc_ (mA/cm^2^)	V_oc_ (mV)	FF (%)	PCE (%)
Planar	7.18 × 10^27^	27	680.5	70.21	12.9
ZrN (50 nm)	7.61 × 10^27^	28.8	681.9	70.48	13.7
ZrN (75 nm)	8.59 × 10^27^	32.6	685.1	70.96	15.7
ZrN (100 nm)	9.04 × 10^27^	34.2	686.4	71.14	16.6
TiN (50 nm)	7.90 × 10^27^	29.71	683.5	70.64	14.3
TiN (75 nm)	9.23 × 10^27^	34.7	687.57	71.23	17
TiN (100 nm)	9.82 × 10^27^	36.91	689.2	71.48	18.2
ZrN/SiO_2_ (90 nm)	9.19 × 10^27^	34.5	687.4	71.2	16.9
ZrN/SiO_2_ (115 nm)	1.07 × 10^28^	40.3	691.4	71.8	20

## Conclusion

In summary, refractory plasmonic materials (RPMs) have been investigated as a promising replacement to noble metals as nano-antennas to boost the photoconversion efficiency of lead-free perovskite solar cells. This was mainly based on the light trapping ability of the BPMs. The use of ZrN leads to a total generation rate enhancement of 25.8% with an increase in the PCE from 12.9% to 17%. On the other hand, replacing ZrN with TiN shows a noticeable enhancement in the total generation rate of 18.2% with a PCE of 18.2%. Tremendous enhancement in the optical and electrical characteristics of the PSCs are observed upon the use of ZrN/SiO_2_ core/shell nanoparticles with a total generation rate enhancement of 55% and an overall PCE of 20%, which is yet to be achieved for any Pb-free PSC. We hope our study opens a new avenue towards the realization of high efficiency Pb-free PSCs.
